# Supplementary material for: Adaptation of the multiplexed CRISPR-Cas13 CARMEN RVP assay for longitudinal detection of respiratory pathogens from air samples
Source: Appl Environ Microbiol. 2026 Mar 26;92(4):e02117-25. doi: 10.1128/aem.02117-25 (PMC13101527; doi:10.1128/aem.02117-25)
Supplement: Figure S1 — Results of lag or lead analysis. [file aem.02117-25-s0001.docx]

A, Lag or lead analysis was performed to compare the frequency of schools positive for matched pathogen targets between nasal swab and air sample data. The shift between time bins (ts) is indicated along the x-axis of the heatmap, with an average of 3-4 days per time bin across the entire 2023-2024 sampling period. Spearman correlation coefficients were calculated across all time shifts and are shown as numbers inside each colored box for the indicated targets on the left-hand side of the heatmap. The color within each box indicates the Spearman correlation coefficient for each comparison, with darker color indicating a higher Spearman r coefficient and lighter color indicating a lower Spearman r. Red outlines around boxes indicate statistically significant comparisons (p≤0.05). B, Lag or lead analysis on the smoothed data was performed to compare the frequency of schools positive for matched pathogen targets between nasal swab and air sample data using a moving average across 3 time shifts. The shift between time bins (ts) is indicated along the x-axis of the heatmap. Spearman correlation coefficients are shown as numbers inside each colored box for the indicated targets on the left-hand side of the heatmap. The color within each box indicates the Spearman correlation coefficient for each comparison; red outlines around boxes indicate statistically significant comparisons (p≤0.05)
